# Impact of xylose epimerase on sugar assimilation and sensing in recombinant *Saccharomyces cerevisiae* carrying different xylose-utilization pathways

**DOI:** 10.1186/s13068-023-02422-z

**Published:** 2023-11-06

**Authors:** Viktor C. Persson, Raquel Perruca Foncillas, Tegan R. Anderes, Clément Ginestet, Marie Gorwa-Grauslund

**Affiliations:** Division of Applied Microbiology, Department of Chemistry, Lund University, Lund, Sweden

**Keywords:** Xylose epimerase, Sugar signaling, Biosensor, Xylose isomerase, Xylose reductase, Xylopyranose, Aldose-1-epimerase, *Saccharomyces cerevisiae*, Anomers, Redox

## Abstract

**Background:**

Over the last decades, many strategies to procure and improve xylose consumption in *Saccharomyces cerevisiae* have been reported. This includes the introduction of efficient xylose-assimilating enzymes, the improvement of xylose transport, or the alteration of the sugar signaling response. However, different strain backgrounds are often used, making it difficult to determine if the findings are transferrable both to other xylose-consuming strains and to other xylose-assimilation pathways. For example, the influence of anomerization rates between α- and β-xylopyranose in pathway optimization and sugar sensing is relatively unexplored.

**Results:**

In this study, we tested the effect of expressing a xylose epimerase in *S. cerevisiae* strains carrying different xylose-consuming routes. First, XIs originating from three different species in isogenic *S. cerevisiae* strains were tested and the XI from *Lachnoclostridium phytofermentans* was found to give the best performance. The benefit of increasing the anomerization rate of xylose by adding a xylose epimerase to the XI strains was confirmed, as higher biomass formation and faster xylose consumption were obtained. However, the impact of xylose epimerase was XI-dependent, indicating that anomer preference may differ from enzyme to enzyme. The addition of the xylose epimerase in xylose reductase/xylitol dehydrogenase (XR/XDH)-carrying strains gave no improvement in xylose assimilation, suggesting that the XR from *Spathaspora passalidarum* had no anomer preference, in contrast to other reported XRs. The reduction in accumulated xylitol that was observed when the xylose epimerase was added may be associated with the upregulation of genes encoding endogenous aldose reductases which could be affected by the anomerization rate. Finally, xylose epimerase addition did not affect the sugar signaling, whereas the type of xylose pathway (XI vs. XR/XDH) did.

**Conclusions:**

Although xylose anomer specificity is often overlooked, the addition of xylose epimerase should be considered as a key engineering step, especially when using the best-performing XI enzyme from *L. phytofermentans*. Additional research into the binding mechanism of xylose is needed to elucidate the enzyme-specific effect and decrease in xylitol accumulation. Finally, the differences in sugar signaling responses between XI and XR/XDH strains indicate that either the redox balance or the growth rate impacts the SNF1/Mig1p sensing pathway.

**Supplementary Information:**

The online version contains supplementary material available at 10.1186/s13068-023-02422-z.

## Background

Xylose epimerase (aldose-1-epimerase; EC 5.1.3.3) is a mutarotase enzyme facilitating the interconversion between α- and β-xylopyranose—the two anomers of D-xylose [1]. The xylose epimerase-encoding gene (*xylM)* was first identified in the xylan-degradation operon of *Lactococcus lactis* [2]. It was located in conjunction with other xylose catabolic genes encoding enzymes such as a xylose isomerase (XI) and a xylulose kinase (XK) [2]. Several studies have indicated that xylose catabolic enzymes, such as xylose reductase (XR) and XI, have a preference for the α-xylopyranose anomer [3–5]. In aqueous solution, the two cyclic anomers exist at an equilibrium of 33% α-xylopyranose to 66% β-xylopyranose [1]. However, during the degradation of the xylan polymer, β-xylopyranose monomers are released almost exclusively (99%) [6]. Although spontaneous anomerization from β-xylopyranose to α-xylopyranose does occur, the rate of the reaction may be limited, causing problems for organisms with rapid xylose assimilation [1, 3]. As such, the expression of a xylose epimerase gene might aid xylose metabolism by increasing the amount of available α-xylopyranose, which could be beneficial in a lignocellulose valorization setting. Indeed, the application of xylose epimerase in an XI-carrying industrial *Saccharomyces cerevisiae* strain has been shown to improve its growth rate [7]; however, whether this improvement was strain/pathway specific or more widely applicable remains unknown.

*S. cerevisiae* is a popular choice for lignocellulose valorization due to its high ethanol productivity and tolerance towards inhibitory compounds [8, 9]. However, to enable efficient fermentation of xylose in this yeast, the following genetic modifications are required: (i) the introduction of a xylose catabolic pathway such as the oxido-reductive xylose reductase/xylitol dehydrogenase (XR/XDH) pathway or the XI pathway, (ii) the upregulation of xylulokinase (XK) and the non-oxidative pentose phosphate pathway genes *TAL1/TKL1*, and (iii) the introduction of xylose-specific transporters (e.g., Gal2^N376Y/M435I^, Gxf1, Xltr1^N326F^) [10–14]. The performance of XI strains can further be optimized, e.g., by choosing XI variants with higher catalytic rates and resistance to xylitol inhibition [8]. For instance, XIs isolated from *Lachnoclostridium phytofermentans* and *Parabacteroides* spp*.* have recently shown improved activity compared to the gold standard *Piromyces* sp. XI [8, 15, 16]. XI activity can be further improved by amplifying the gene copy number via, e.g., evolutionary engineering [17] and by deletion of the endogenous aldose reductase gene *GRE3*, whose enzyme reduces xylose into the inhibitory intermediate xylitol [8]. However, despite the above-mentioned genetic engineering, xylose utilization rates and ethanol productivity remain relatively low as compared to glucose. This subpar performance is thought to be caused in part by transport competition between xylose and glucose, and in part by global regulatory changes imparted via carbon catabolite repression from glucose [18].

Carbon catabolite repression is a regulatory response which enables cells to prioritize the catabolism of certain sugars over others [18, 19]. This regulation is controlled by several cross-talking sugar signaling pathways, which monitor nutrients both intra- and extracellularly [18]. Overall, the sugar signaling of *S. cerevisiae* in response to glucose is very well-studied, but much remains unclear about the responses to other sugars such as xylose [18]. Earlier studies have indicated that non-engineered *S. cerevisiae* fails to recognize xylose as fermentable carbon source, as seen by the lack of activation in the sugar signaling pathways when xylose is present [20]. Incorporation of the XR/XDH pathway allowed the cells to partially activate the sugar signaling pathways, evident from the induction of *SUC2*p which is under the control of the SNF1/Mig1p nutrient sensing pathway [21]. Most likely, the intracellular metabolites responsible for this activation are glycolytic intermediates such as fructose-6-phosphate [22]. However, the potential redox imbalance in XR/XDH strains, caused by the promiscuous co-factor preference of XR, may also influence the SNF1/Mig1p sugar signaling pathway via crosstalk from redox-sensitive pathways. By comparing response from the redox neutral XI strains to the XR/XDH strain, it may be possible to tell if the redox balance is part of triggering the sugar signaling response on xylose or whether it results from other common intracellular metabolites. This, in turn, may lead to new engineering targets for improving xylose utilization.

In this study, the effect of expressing xylose epimerase in strains optimized for xylose utilization carrying either the XR/XDH pathway or different variants of the XI pathway were investigated. The growth rates, xylose utilization and xylitol byproduct formation were analyzed under aerobic conditions to identify which xylose catabolizing enzymes benefited the most from xylose epimerase introduction. The strains with the best-performing enzymes were further compared and analyzed under anaerobic conditions. Furthermore, the sugar signaling responses of the newly created XI strains were compared to those of the established XR/XDH strains.

## Result

### Effect of xylose epimerase on three different xylose isomerases

After the successful expression of the xylose isomerase (XI) from *Piromyces* sp. (PiroXI) in *S. cerevisiae* [23], the search for even more efficient enzymes has continued. Two alternative XI candidates of particular interest have emerged: (i) ClosXI (discovered in *Lachnoclostridium phytofermentans*) which has been further modified to improve activity and avoid xylitol inhibition [15], and (ii) ParaXI (recently isolated from *Parabacteroides* spp.) which has a higher activity than PiroXI and whose gene expression in *S. cerevisiae* has resulted in faster growth on xylose [16]. In the present study, the three variants (PiroXI, ClosXI and ParaXI) were directly compared for the first time in an isogenic W303 strain background. The W303 background was preferred over the CEN.PK background because the latter carries mutations in the main sugar signaling pathways that can affect the result of sensing studies [18]. The strains were modified with the overexpression of the *TAL1* and *TKL1* genes from the pentose phosphate pathway, and deletion of the endogenous aldose reductase *GRE3* gene to avoid xylitol formation, a potent inhibitor of XIs. The effect of introducing a xylose epimerase, an aldose-1-epimerase catalyzing the anomerization between α-xylopyranose and β-xylopyranose, was also studied for each XI variant. To ensure that the differences observed between the strains were linked to the efficiency of the genes rather than their expression levels, the same promoter was used for all XI variants. Furthermore, to achieve a similar copy number of the genes, the same chromosome loci were used for the integration of two copies and the same multicopy plasmid chassis was used in all strains (see Methods).

**Fig. 1:**
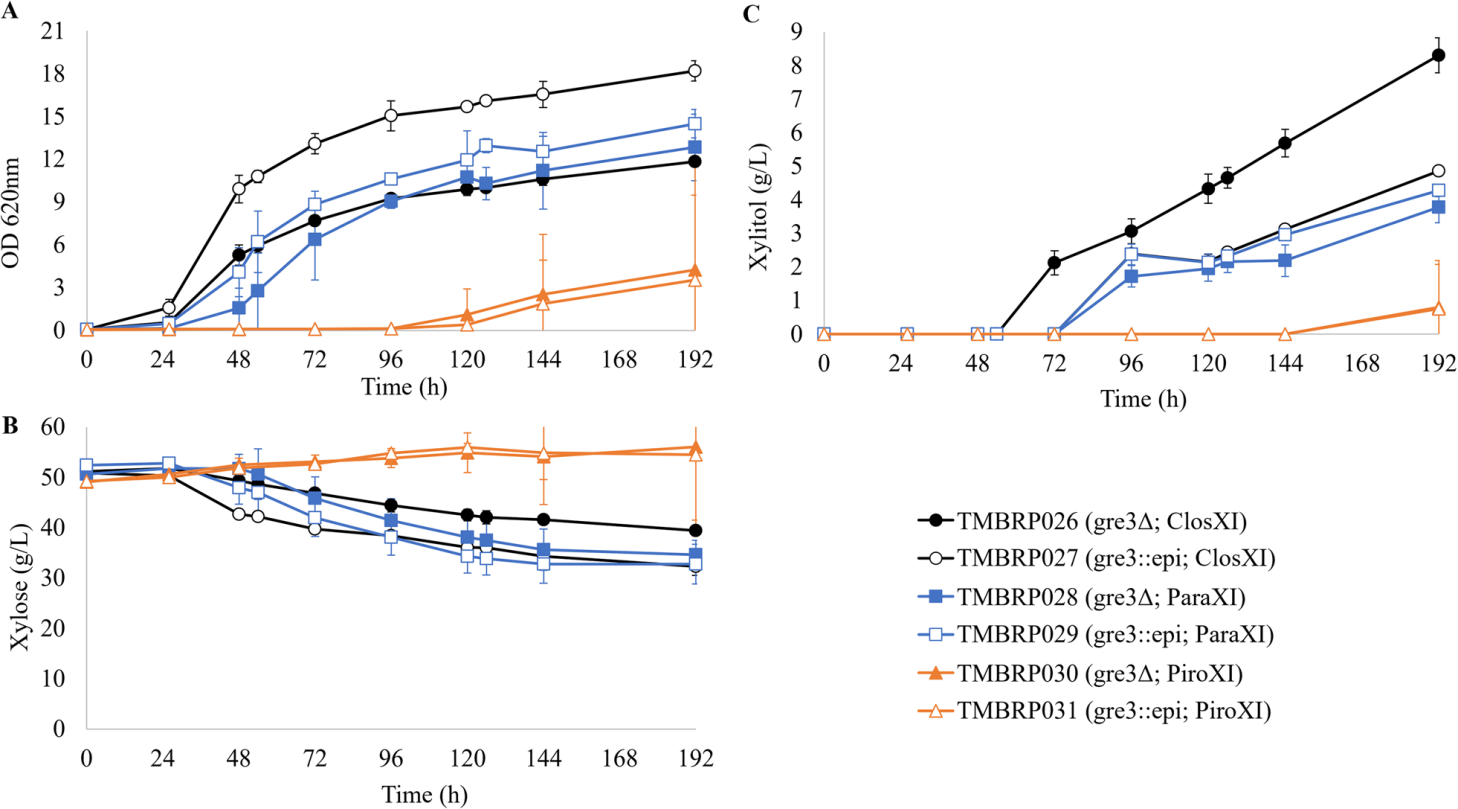
Aerobic cultivation of XI strains. **A** Optical density at 620nm, **B** xylose concentration and **C** xylitol concentration over time during aerobic cultivation in 250-mL baffled shake flasks containing YNB medium supplemented with 50 g L^−1^ xylose. At least two biological replicates were performed

The strains carrying either ClosXI (TMBRP026-027) or ParaXI (TMBRP028-029) started growing after 24 h, whereas the strains carrying PiroXI (TMBRP030-031) showed a much longer lag phase with growth only after 120 h (Fig. 1A). The results were in agreement with the observed xylose consumption profiles (Fig. 1B). The introduction of the xylose epimerase considerably impacted the performances in the case of ClosXI, where higher biomass and faster xylose consumption were observed (Fig. 1A, B); this was associated with a 14% increase in the maximum specific growth rate (Additional file 1: Table S1). The positive impact was less striking with ParaXI, where only a slight reduction in lag phase and no significant change in maximum specific growth rate could be observed (Fig. 1A, Additional file 1: Table S1). Finally, the introduction of xylose epimerase had no visible impact in the poorly growing PiroXI-carrying strain. Curiously, for the best XI candidate (ClosXI), the levels of xylitol produced were reduced by the introduction of the xylose epimerase (Fig. 1C).

### Effect of xylose epimerase on anaerobic xylose fermentation in strains carrying XI vs XR-XDH pathways

To investigate whether the epimerization of xylose also had a positive impact on strains carrying the oxido-reductive xylose reductase/xylitol dehydrogenase (XR/XDH) pathway, isogenic strains with the *GRE3* deletion and xylose epimerase gene integration were constructed. This yielded the strains TMBRP024 (gre3Δ, XR/XDH) and TMBRP025 (gre3::epimerase, XR/XDH) that were compared to the XI strains that best performed under aerobic conditions (ClosXI; TMBRP026 and TMBRP027) and to control strains lacking any of the xylose assimilation pathways (TMBVP1005, TMBVP1105, Table 2).

**Fig. 2:**
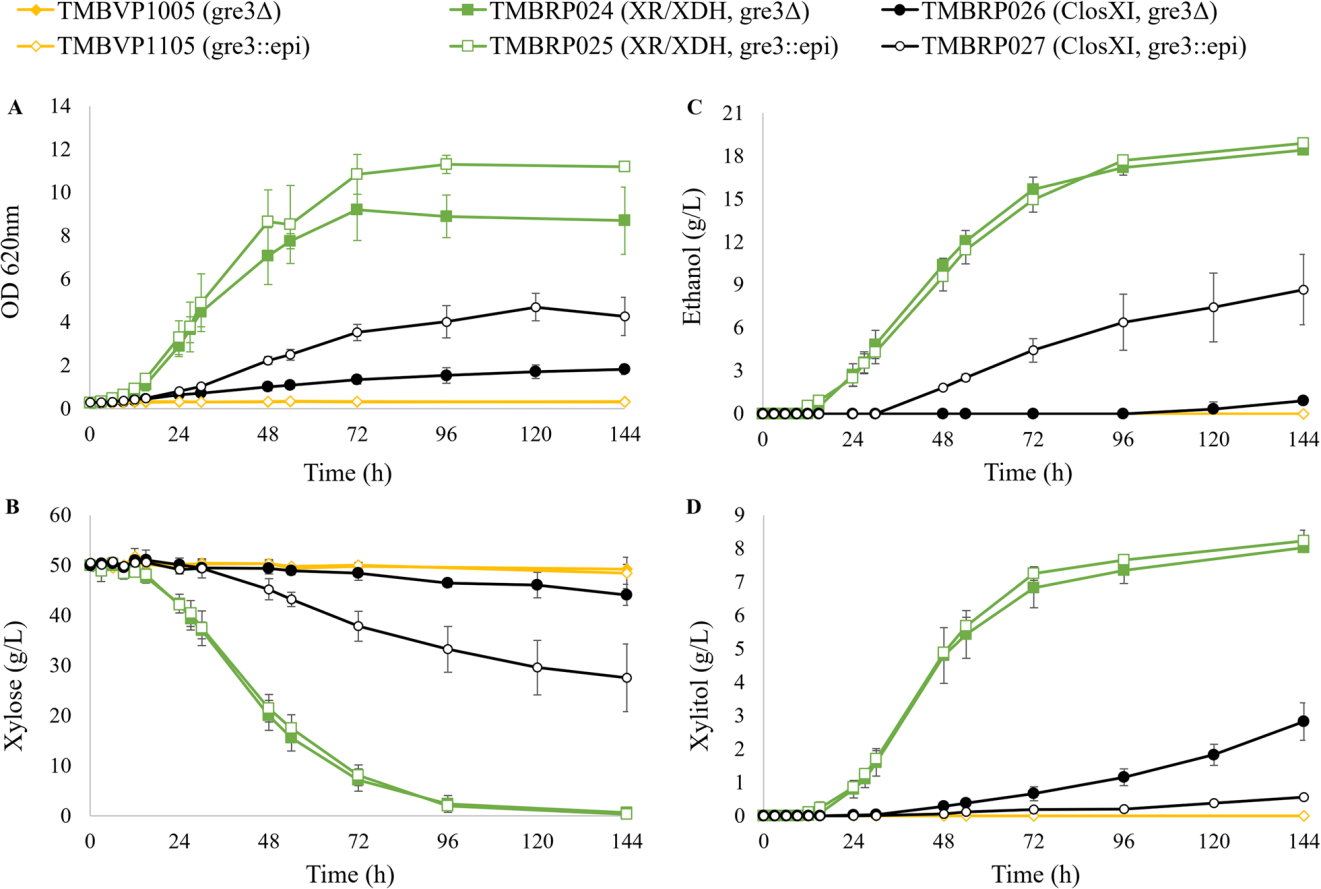
Anaerobic cultivation of XI, XR/XDH and control strains. **A** Optical density at 620nm, **B** xylose concentration, **C** ethanol concentration and **D** xylitol concentration over time during anaerobic cultivation in serum vials containing YNB medium supplemented with 50 g L^−1^ xylose. Biological duplicates were performed. ClosXI: XI from *Lachnoclostridium phytofermentans*, epi: xylose epimerase

As anticipated, the strains lacking a catabolic pathway for xylose (TMBVP1005 and TMBVP1105) were incapable of growth or consumption of xylose as the sole carbon source (Fig. 2A, B). Of the strains growing on xylose, those containing the XR/XDH pathway exhibited superior growth compared to those carrying the XI pathway (Fig. 2A).

Among the two ClosXI strains, the one carrying xylose epimerase (TMBRP027) reached higher OD, had better xylose consumption rate, reduced xylitol accumulation, and increased ethanol production (Fig. 2) and yields (Additional file 1: Table S2). These results confirmed that the introduction of xylose epimerase in ClosXI-carrying strains has a positive effect under anaerobic conditions as well. The elevated OD was further corroborated by an increase in cell dry weight (Additional file 1: Fig. S1).

Among the two XR/XDH strains, the xylose epimerase strain (TMBRP025) reached a slightly higher OD than its epimerase-less counterpart (TMBRP024) (Fig. 2A). Nevertheless, this difference in growth was not accompanied by any change in xylose consumption, xylitol accumulation nor ethanol formation (Fig. 2B, C, D). Further analysis of the strain growth revealed that the difference observed in OD did not correspond to an actual difference in cell dry weight (Additional file 1: Fig. S1), suggesting there is no positive effect of integrating xylose epimerase in the investigated XR/XDH strains.

### Effect of *GRE3* deletion and xylose epimerase in XR/XDH strains

The expression level of the *GRE3* gene, encoding an endogenous aldose reductase, has been demonstrated to impact the ethanol production from xylose. However, the final outcomes of *GRE3* engineering in XR/XDH strains are inconsistent between studies. While overexpression of *GRE3* in an XR/XDH background did not benefit a laboratory strain grown on xylose [24], it did enhance ethanol yield in an industrial strain fermenting lignocellulosic hydrolysates [25]. One potential explanation for this inconsistency is the established capability of Gre3p to detoxify furfural and HMF, which may have been present in the hydrolysate sample [26]. The deletion of the *GRE3* gene has also been reported to have a negative impact on the biomass yield in a XR/XDH background. Its deletion, however, was associated with an increased ethanol yield and a decreased xylitol yield from xylose [24]. Therefore, since *GRE3* had been used as the target locus of xylose epimerase in this study, we decided to reassess the effect of *GRE3* deletion in the XR/XDH background. A new XR/XDH strain carrying the xylose epimerase alongside an intact *GRE3* (TMBRP033) was constructed by instead targeting the epimerase gene integration to an intergenic locus on chromosome X. The strain was compared with the background strain TMB3755 (XR/XDH) as well as the two previously described TMBRP024 (gre3Δ, XR/XDH) and TMBRP025 (gre3::epimerase, XR/XDH), with the objective to distinguish between the effect of the two modifications (*GRE3* deletion *vs.* epimerase expression) in the XR/XDH background. The strains were evaluated under aerobic conditions in YNB medium supplemented with 50 g L^−1^ xylose.

**Fig. 3:**
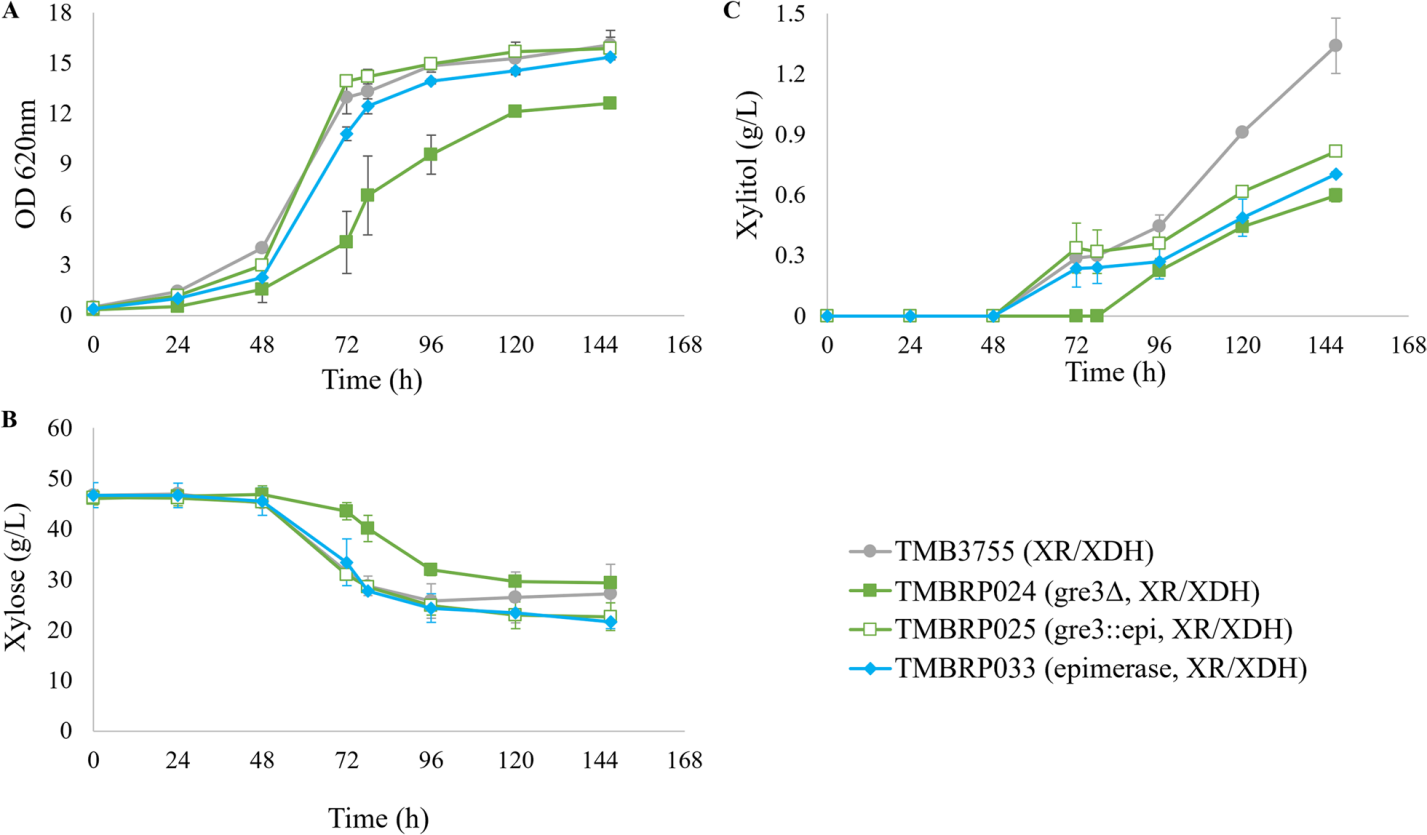
Aerobic cultivation of XR/XDH strains. **A** Optical density at 620nm, **B** xylose concentration and **C** xylitol concentration over time during aerobic cultivation in 250-mL baffled shake flasks containing YNB medium supplemented with 50 g L^−1^ xylose. Biological duplicates were performed. epi: xylose epimerase

An impairment in growth and xylose consumption was confirmed with *GRE3* gene deletion, as TMBRP024 strain (gre3Δ, XR/XDH) displayed slower and reduced growth as compared to the background strain TMB3755 (XR/XDH) (Fig. 3A, B). The same effect was not observed in the strain carrying *GRE3* deletion in combination with the epimerase expression, TMBRP025 (gre3::epimerase, XR/XDH) (Fig. 3A, B). In that strain, growth was not impaired, indicating a putative beneficial effect of the xylose epimerase that would counterbalance the negative impact of *GRE3* deletion. However, the sole addition of the xylose epimerase (TMBRP033 (epimerase, XR/XDH)) did not improve growth on xylose as compared to the background strain TMB3755 (XR/XDH).

To determine if the *GRE3*-rescuing phenotype observed in TMBRP025 (gre3::epimerase, XR/XDH) was directly connected to xylose metabolism or whether the addition of the epimerase had a generalized effect on cellular metabolism, the strains were grown in YNB supplemented with 20 g L^−1^ glucose instead. When glucose was used as carbon source, no growth differences were observed between the strains (Additional file 1: Fig. S2). This indicates both that *GRE3* deletion did not lead to growth impairment on glucose and that the rescuing effect of the deletion of *GRE3* by the epimerase was xylose-specific.

At the product level, a decrease in the amount of accumulated xylitol was observed in the XR/XDH strains containing the xylose epimerase, regardless of the presence or absence of the *GRE3* gene (Fig. 3C), similar to the decreased xylitol production found for the ClosXI route.

### Impact of xylose epimerase and xylose pathway on sugar sensing

To determine whether the sugar sensing response was impacted by the inclusion of xylose epimerase or by the type of implemented xylose assimilation pathway, a transcription-based green fluorescent protein (GFP) biosensor for the *SUC2* promoter (*SUC2*p-*yEGFP*) was used. This biosensor was included in all strains carrying the multicopy XR/XDH or XI pathways. Using flow cytometry, the biosensor enabled monitoring of the sugar signaling response of the SNF1/Mig1p pathway over time on xylose.

**Fig. 4:**
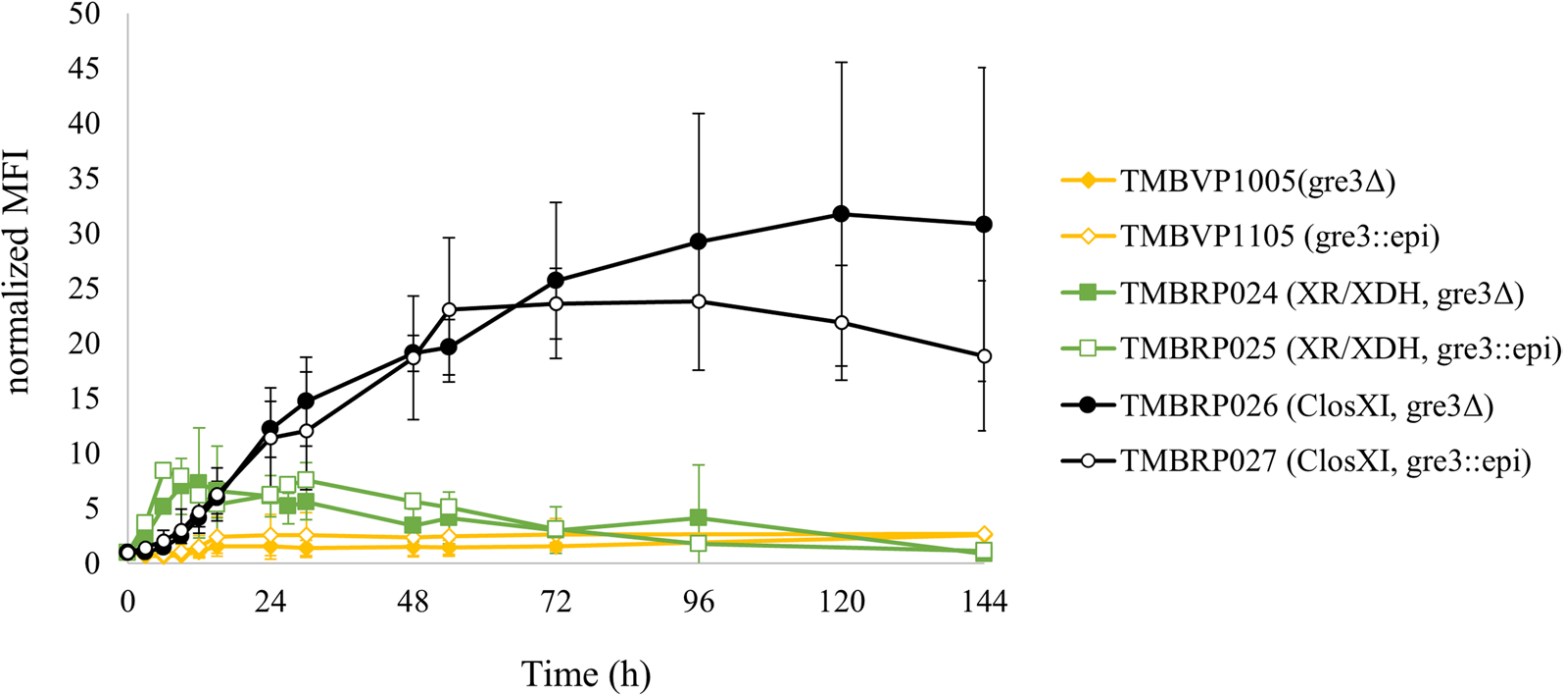
Sugar signaling response of XI, XR/XDH and control strains. Normalized mean fluorescence intensity of the *SUC2*p-*yEGFP* biosensor over time during anaerobic cultivation in YNB medium supplemented with 50 g L^−1^ xylose. Biological duplicates were performed. ClosXI: XI from *Lachnoclostridium phytofermentans*, epi: xylose epimerase

The addition of the xylose epimerase did not significantly influence the signal of the *SUC2*p-*yEGFP* sensor regardless of the studied strain under anaerobic conditions (Fig. 4). Also, no fluorescence was observed from the *SUC2*p-*yEGFP* biosensor in the strains lacking a pathway for xylose assimilation (Fig. 4). This confirmed earlier results indicating that the presence of external xylose was not enough to generate a sugar signaling response and that xylose had to be internalized [27], but also indicated that changes in anomer balance had no effect on the (lack of) external xylose sensing.

Interestingly, between the XI and XR/XDH strains significantly different signal responses were observed. The faster-growing strains carrying the XR/XDH pathway showed an initial increase in fluorescence during the first 9 to 12 h where it peaked and slowly decreased over time. In the case of the XI strains, a much slower initial fluorescence response was observed. Nevertheless, the fluorescence increased gradually until 48 h and 120 h for TMBRP027 (gre3::epimerase; ClosXI) and TMBRP026 (gre3Δ; ClosXI), respectively. A plateau was reached in both cases afterwards.

## Discussion

Improvement of xylose utilization in *S. cerevisiae* has been the topic of a wide number of studies in the last decades. However, there are very few attempts to compare parallel strategies in the same strain background and setup. This is what we did in the present study, where the objective was to answer the following questions: How do the currently most promising XIs compare in the same isogenic strain? Does xylose epimerase affect all XIs in the same manner? Does xylose epimerase expression also benefit xylose utilization by the XR-XDH route? What is the impact of *GRE3* deletion in both routes? Does the ratio between xylose anomers affect xylose sensing? Are there differences in sugar signaling when a redox neutral or cofactor-imbalanced xylose pathways are expressed?

Strains carrying XI variants are often selected after successive rounds of adaptation in xylose-containing solid and liquid media [28]; this leads to amplification of the XI gene cassette and makes it difficult to compare XI enzyme efficiency per se. In our approach, where no adaptation was applied, ClosXI clearly was the most efficient candidate of the three tested enzymes, with similar growth rate and xylose consumption as ParaXI but shorter lag phase. Also, no reliable growth was achieved in the strains carrying the well-established XI from *Piromyces* sp., PiroXI, confirming the key role of adaptation. It cannot be ruled out that the use of a multicopy plasmid, in addition to the double genome integration, influenced our results, but very similar results between clones of the same constructs (data not shown) go against this hypothesis.

The beneficial role of xylose epimerase on xylose utilization by XI enzymes has previously been demonstrated in an industrial XI-carrying strain background [7]. However, differences in the extent of improvement were already observed when using either the XI from *Piromyces* sp. or the XI from *Thermoanaerobacter thermohydrosulfuricus* [7]. A similar result was observed here in the tested laboratory strain background W303 with a much more significant impact of the epimerase on ClosXI than on ParaXI. The positive impact observed under aerobic conditions was confirmed during anaerobic growth, with ethanol yields increasing from 0.18 g/g xylose (TMBRP026; gre3Δ, ClosXI) to 0.38 g/g xylose after the addition of the epimerase (TMBRP027; gre3::epimerase, ClosXI). Since spontaneous conversion between the two xylose anomers is possible, the benefit of xylose epimerase expression is only expected in strains where low anomerization rates constitute the primary bottleneck for efficient xylose consumption. This would likely only occur in strains with very high xylose consumption rates, and with catabolic enzymes that have a preference for one anomer over the other. Consequently, the beneficial effects of xylose epimerase observed for ClosXI suggests that the enzyme is not only very rapid, but that it has a preference for one anomer over the other as previously reported for other XIs [4]. To test this anomer specificity, one could monitor for anomer depletion via nuclear magnetic resonance (NMR) at low temperatures as demonstrated by Vogl and colleagues [3]. Alternatively, one could model the 3D structure of the xylose isomerase and analyze the molecular docking of the two xylose anomers into the catalytic pocket. Unfortunately, no experimental 3D structures are available neither for ClosXI nor ParaXI, and our attempts at molecular docking using AlphaFold2-derived structures proved unreliable as even minute changes in protein folding could extensively alter binding affinities (data not shown).

No significant effect of xylose anomerization was observed in the strains carrying the alternative xylose assimilation pathway (XR/XDH) indicating either that xylose anomerization is not a rate-controlling step or that the XR used in this study, isolated from *Spathaspora passalidarum* (SpXR), does not have anomer specificity. An exact mechanism has yet to be determined for XR catalysis, however there are generally two views: either catalysis occurs by binding of XR to the linear xylose molecules and “trapping” the relatively low fraction of linear xylose available, or binding occurs directly to the cyclic xylopyranose molecules which is followed by ring-opening [29]. The former view is largely based on manual docking attempt of XR to linear xylose [30, 31], and on the behavior of glucose reductase isolated from pig which seem to bind and trap the relatively low fraction of linear glucose in enzymatic assays [32, 33]. This mechanism would validate why SpXR does not show any anomer specificity. However, another study investigating *Candida tenuis* XR (CtXR) substrate-binding affinity revealed a preference for the cyclic α-xylopyranose over other xylose fractions, directly supporting a cyclic xylose-binding mechanism [3]. This mode of catalysis, binding of cyclic α-xylopyranose followed by ring-opening, would be the same as suggested to be employed by XI enzymes from, e.g., *Streptomyces* sp. and *Arthrobacter* sp*.* [4, 5]. If the binding of cyclic xylose is a general behavior of XRs, then the lack of effect from xylose epimerase in our SpXR/XDH strains is likely because the catabolic pathway is limited in some other steps. Further studies on anomer specificity in XRs would help determine if the mechanism of CtXR is the norm, or if other XR enzymes have different anomer preferences. Expression of XRs with a specific anomer preference, or lack thereof, may be a way to further improve lignocellulosic bioprocesses.

Quite unexpectedly, a decrease in the amount of produced xylitol was observed in the strains carrying the xylose epimerase. Since the primary aldose reductase *GRE3* was deleted in the strain TMBRP027 (gre3::epimerase, ClosXI), other endogenous reductases capable of transforming xylose into xylitol are expected to be responsible for this xylitol production [34]. Consequently, the decrease observed in TMBRP027 could be a result of these other aldose reductases having an anomer preference. Alternatively, faster consumption by the xylose isomerase could lead to a lower availability of xylose for these reductases. Regardless of the mechanism behind it, limiting xylitol formation in XI-based strains is a beneficial trait and further points towards xylose epimerase as a useful addition. For the XR/XDH strains, the decrease in xylitol accumulation was only observed under aerobic conditions. Although SpXR is the main enzyme responsible for the xylitol formation in these strains, it is known that xylose conversion via XR enzymes which have increased preference for NADH is less efficient under aerobic conditions than under anaerobic conditions, likely due to a NAD^+^ shortage due to the competitive respiration route [35]. As such, the role of other endogenous reductases might be more pronounced during aerobic growth; so if these other reductases have an anomer preference it might explain why the lower formation of xylitol was only visible under aerobic conditions.

In previous work, the addition of the XR/XDH pathway caused a change to the sugar signaling pathways of *S. cerevisiae*, consistent with sensing xylose as a poor—yet fermentable—carbon source [20, 21]. However, it remained unclear whether this xylose signaling response was specific to XR/XDH strains or whether it also occurred in XI strains. The present study aimed at answering this question by exploring the sugar signal response in both XR/XDH and XI strains using a transcription-based biosensor targeting *SUC2*p, a promoter activated by the SNF1/Mig1p signaling pathway primarily upon nutrient limitation [18, 27]. Whereas previous studies focused on a single time-point fluorescence signal after 6 h of cultivation, the present study monitored fluorescence throughout the cultivation, thereby giving additional information on the sensing kinetics. It revealed a significantly different signaling pattern between strains carrying the two xylose pathways, with XI strains displaying both a higher and more prolonged induction of the *SUC2*p biosensor. For the XR/XDH strains, an initial induction of the sensor was observed, in agreement with previously reported data [21]. Interestingly, it also showed that after the initial peak, the induction levels of the sensor decreased over time—which could indicate that the nutrient limitation signal observed on xylose was transient. In the case of XI strains, on the other hand, the fluorescence increased continuously over time reaching much higher induction levels than those of XR/XDH strains. In general, increased induction of *SUC2* indicates that the cell is detecting a lack of fermentable sugars, which results in global regulatory changes of gene expression that may decrease xylose utilization rates [18]. This corroborates the poorer growth of the XI strains as compared to the XR/XDH strain. Two main differences exist between the XR/XDH and XI pathways in our setup: (i) the level of xylose metabolizing enzymes and their efficiency, and (ii) the redox balance of the cell since assimilation of xylose via the XR/XDH route is not cofactor balanced [8]. Although the observed difference in sugar signaling between XR/XDH and XI strains could speak in favor of the redox imbalance impacting the sugar signaling response, the considerably faster growth rate of XR/XDH strains is also likely to influence the response. With an increased growth rate and xylose consumption, intracellular intermediate levels and glycolytic fluxes are increased which may impact the sugar signaling of SNF1/Mig1p. Moreover, culture conditions such as total sugar concentration decrease at a quicker rate and other cross-talking signaling pathways may act to influence the results. Thus it is still difficult to conclusively determine how large the impact of the redox imbalance is on the difference in *SUC2*p induction between the strains. To further answer this question, the differences in growth rate have to be eliminated, for example, by cultivating both the XR/XDH and XI strains in chemostat cultivations with the same dilution rate.

## Conclusion

In this study, three different XI variants, ClosXI, ParaXI and PiroXI were compared for the first time in isogenic, non-evolved strains of *Saccharomyces cerevisiae*. ClosXI was determined to be the most efficient XI candidate among the three tested XIs. The effect of introducing a xylose epimerase into xylose-consuming strains was shown to be enzyme-dependent, but it was confirmed that its addition was beneficial and led to better xylose consumption rate, lower xylitol accumulation and higher ethanol production. This was the case for the strain carrying ClosXI, the strain which showed the highest xylose consumption and hence the one most susceptible to being bottlenecked by the anomerization rate. On the other hand, strains carrying SpXR showed no improvement by the presence of a xylose epimerase, suggesting that SpXR might not have anomer preference. Using the biosensor *SUC2*p-*yEGFP* to monitor the sugar signaling response, we showed that xylose epimerase has no impact on xylose sensing. However, significant differences were observed both in pattern and magnitude of the fluorescence response between XI and XR/XDH strains, indicating that difference in redox balancing may influence the SNF1/Mig1p pathway, although growth rate difference still needs to be ruled out as an alternative cause.

## Methods

### Strains and cultivation media

All shuttle plasmids and yeast strains used and developed in this study are listed in Table 1 and Table 2, respectively.

**Table 1:** List of plasmids used in this study

Plasmid name	Relevant genotype	References
pCfB2312	*TEF1*p-*Cas9*-*CYC1*t; kanMX	[38]
pCfB2899	X-2 MarkerFree backbone	[38]
pCfB3037	XI-5-MarkerFree backbone	[38]
pCfB3040	XII-4- MarkerFree backbone	[38]
pCfB3020	gRNA_X-2; natMX	[38]
pCfB3046	gRNA_XI-5; natMX	[38]
pCfB3049	gRNA_XII-4; natMX	[38]
pCfB3053	gRNA_X-2; gRNA_XI-5; gRNA_XII-4; natMX	[38]
pLWA25	gRNA_GRE3; natMX	[39]
pLWA31	pCfB3037; *TDH3*p-*SpXYL1.2*-*ADH1*t, *TEF1*p-*SsXYL2*-*GPM1*t, *PGI1*p-*XKS1*-*PYK1*t	[21]
pLWA33	pCfB3040; *TDH3*p-*SpXYL1.2*-*ADH1*t, *TEF1*p-*SsXYL2*-*GPM1*t, *PGI1*p-*XKS1*-*PYK1*t	[21]
YEpHXT_PiroXI	pHXT7; HXT7^*^p-*xylA (Piromyces)*-*PGK1*t; *URA3*	[14]
pRS42N	*TEF1*p-*natMX*-*ADH1*t	[40]
pVP001	pUC57; *PGK1*p-*xylM*-*CYC1*t	This study
pUC57 + ClosXI	pUC57; *TEF1*p- *LpXImut2* -*GPM1*t	This study
pUC57 + ParaXI	pUC57; *xylA (Parabacteroides)*	This study
pRP021	pCfB2899; *TEF1*p-*LpXImut2*-*GPM1*t; *PGI1*p-*XKS1*-*PYK1*t	This study
pRP023	pCfB3037; *TEF1*p-*LpXImut2*-*GPM1*t; *PGI1*p-*XKS1*-*PYK1*t	This study
pRP024	pCfB2899; *TEF1*p-*xylA(Parabacteroides)*-*GPM1*t; *PGI1*p-*XKS1*-*PYK1*t	This study
pRP026	pCfB3037; *TEF1*p-*xylA(Parabacteroides)*-*GPM1*t; *PGI1*p-*XKS1*-*PYK1*t	This study
pRP027	pCfB2899; *TEF1*p-*xylA(Piromyces)*-*GPM1*t; *PGI1*p-*XKS1*-*PYK1*t	This study
pRP029	pCfB3037; *TEF1*p-*xylA(Piromyces)*-*GPM1*t; *PGI1*p-*XKS1*-*PYK1*t	This study
pRP032	pRS42N; *TEF1*p-*LpXImut2*-*GPM1*t	This study
pRP033	pRS42N; *TEF1*p-*xylA (Parabacteroides)*-*GPM1*t	This study
pRP034	pRS42N; *TEF1*p-*xylA (Piromyces)*-*GPM1*t	This study

^*^The truncated *HXT7* promoter, consisting only of the 390bp upstream of the start codon

**Table 2:** List of *S. cerevisiae* strains used in this study

Strain name	Relevant genotype	References
TMB3700	W303-1A *TRP1 HIS3*; *ura3*::M3499 (*ADE2*)	[27]
TMB3725	TMB3700; *can1*:: *SUC2*p-*yEGFP*-*PGK1*t; SPB1/PBN1::YIp128*GAL2*mut	[21]
TMB3745	TMB3725; Vac17/MRC1::*FBA1*p*-TKL1-PDC1*t, *TPI1*p*-TAL1-CPS1*t; pCfB2312	This study
TMB3755	TMB3725; pCfB2312, Vac17/MRC1::*FBA1*p*-TKL1-PDC1*t, *TPI1*p*-TAL1-CPS1*t; Chr X-2/XI-5/XII-4:: *TDH3*p*-SpXYL1.2-ADH1*t*, TEF1*p*-SsXYL2-GPM1*t*, PGI1*p*-XKS1-PYK1*t	[21]
TMBVP1005	TMB3745; *gre3*Δ	This study
TMBVP1105	TMB3745; gre*3*::*PGK1*p-*xylM*-*CYC1*t	This study
TMBRP016	TMBVP1005; X-2/XI-5::*TEF1*p-*LpXImut2*-*GPM1*t-*PGI1*p-*XKS1*-*PYK1*t	This study
TMBRP017	TMBVP1105; X-2/XI-5::*TEF1*p-*LpXImut2*-*GPM1*t-*PGI1*p-*XKS1*-*PYK1*t	This study
TMBRP018	TMBVP1005; X-2/XI-5::*TEF1*p-*xylA (Piromyces)*-*GPM1*t-*PGI1*p-*XKS1*-*PYK1*t	This study
TMBRP019	TMBVP1105; X-2/XI-5::*TEF1*p-*xylA (Piromyces)*-*GPM1*t-*PGI1*p-*XKS1*-*PYK1*t	This study
TMBRP020	TMBVP1005; X-2/XI-5::*TEF1*p-*xylA (Parabacteroides)*-*GPM1*t-*PGI1*p-*XKS1*-*PYK1*t	This study
TMBRP021	TMBVP1105; X-2/XI-5::*TEF1*p-*xylA (Parabacteroides)*-*GPM1*t-*PGI1*p-*XKS1*-*PYK1*t	This study
TMBRP024	TMBVP1005; X-2/XI-5/XII-4::*TDH3*p*-SpXYL1.2-ADH1*t*, TEF1*p*-SsXYL2-GPM1*t*, PGI1*p*-XKS1-PYK1*t	This study
TMBRP025	TMBVP1105; X-2/XI-5/XII-4::*TDH3*p*-SpXYL1.2-ADH1*t*, TEF1*p*-SsXYL2-GPM1*t*, PGI1*p*-XKS1-PYK1*t	This study
TMBRP026	TMBRP016; pRP032	This study
TMBRP027	TMBRP017; pRP032	This study
TMBRP028	TMBRP018; pRP033	This study
TMBRP029	TMBRP019; pRP033	This study
TMBRP030	TMBRP020; pRP034	This study
TMBRP031	TMBRP021; pRP034	This study
TMBRP033	TMB3755; X-4::*PGK1*p-*xylM*-*CYC1*t	This study

*Escherichia coli* NEB5α competent cells from New England Biolabs (Ipswich, MA, USA) were used for sub-cloning purposes. Liquid cultures of *E. coli* were performed in Lysogeny Broth (LB) medium containing 10 g L^−1^ tryptone, 5 g L^−1^ yeast extract, 5 g L^−1^ NaCl, pH 7.0. Selection of successful transformants was done in LB agar plates (LB + 15 g L^−1^ agar) supplemented with ampicillin (50 mg L^−1^) and incubated overnight at 37 °C.

For yeast transformations, *Saccharomyces cerevisiae* strains were grown in Yeast Peptone Dextrose (YPD) medium containing 20 g L^−1^ peptone, 10 g L^−1^ yeast extract and 20 g L^−1^ glucose. Selection of transformants was done at 30 °C in YPD agar plates (YPD + 15 g L^−1^ agar) supplemented with geneticin (200 mg L^−1^) and nourseothricin (100 mg L^−1^) to select for the Cas9-kanMX and the gRNA-natMX plasmids, respectively.

For strain characterization experiments, the defined mineral medium Yeast Nitrogen Base (YNB) was used and supplemented with 20 g L^−1^ glucose or 50 g L^−1^ xylose as carbon source.

All yeast cultivations were performed at 30 °C and 180 rpm in a rotary shake incubator (Innova 43, New Brunswick Scientific Co. Inc., NJ, USA).

### Plasmid construction

The sequence for an aldose-1-epimerase gene (*xylM*) identified in the xylan-degradation operon of *Lactococcus lactis* [36] was codon-optimized for yeast [37] and de novo synthesized on a pUC57 shuttle plasmid containing the *CYC1*t terminator and the strong constitutive promoter *PGK1*p by GenScript (Piscataway, NJ, United States). The plasmid was named pVP001 and was maintained and subcloned in *E. coli*.

The sequences for the genes encoding the xylose isomerase from *Lachnoclostridium phytofermentans* (ClosXI) and from *Parabacteroides* spp. (ParaXI) were obtained from Kobayashi et al. and Da Silva et al., respectively [15, 16]. They were codon-optimized for *Saccharomyces cerevisiae* [37] and de novo synthesized by GenScript (Piscataway, NJ, United States). These plasmids were named pUC57 + ClosXI and pUC57 + ParaXI. The construct *TEF1*p*-ClosXI* was amplified from pUC57 + ClosXI using the primers *TEF1p_AscI_f* and *XI-XK_OE_PCR_r* (Additional file 1: Table S3). The PCR product was digested with DpnI and ligated into pLWA32 and pLWA31 using AscI/MreI restriction sites. The plasmids obtained were called pRP021 and pRP023, respectively, and served as backbone for the generation of plasmids containing different XI variants by replacing the XI-encoding gene using the SfaAI/MreI restriction sites. The gene encoding ParaXI was directly digested with SfaAI/MreI from pUC57 + ParaXI. In the case of PiroXI, the coding region was amplified from YEpHXT_PiroXI using the primers *PiroXI_SfaAI_f* and *PiroXI_MreI_r* (Additional file 1: Table S3) and digested with SfaAI/MreI.

The multicopy plasmids containing the different XI gene variants were obtained by ligation of the corresponding insert into pRS42N using BamHI/NotI restriction sites. The insert used to obtain pRP032, *TEF1p-ClosXI-GPM1t*, was amplified from pRP021 with primers *TEF1p_BamHI* and *GPM1t_NotI* (Additional file 1: Table S3). The same primers were used to amplify the construct *TEF1p-ParaXI-GPM1t* from pRP024 and *TEF1p-PiroXI-GPM1t* from pRP027 to obtain pRP033 and pRP034, respectively.

### Strain construction

The yeast strains were engineered using a CRISPR/Cas9 system with double homologous recombination of various linear donor DNAs [38]. The TMB3725 strain was initially transformed with pCfB2312 to express the Cas9 activity. Afterwards, the *TKL1-TAL1* fragment was integrated in the *VAC17/MRC1* intergenic region by using the pLWA19 plasmid linearized with SfaAI and NotI as donor DNA and the plasmid pLWA26 for the gRNA as previously described [21]. This process yielded the background strain TMB3745.

#### *GRE3* deletion

To obtain the *GRE3*-deletant strain TMBVP1005, the background strain TMB3745 was transformed with pLWA25 and two short homologous overlap fragments (456 bp and 364 bp, respectively) which were generated by PCR and overlapped both the *GRE3* region and each other. The primers used to create the fragments were *v009_GRE3_DS_Blank* together with *87R,* and *84F* together with *v008_GRE3_US_Blank* (Additional file 1: Table S3). The resulting triple-fragment double homologous recombination led to the deletion of *GRE3*.

#### Integration of xylose epimerase in *GRE3* locus

The strain TMBVP1105 containing the aldose-1-epimerase gene (*xylM*) in the *GRE3* region was obtained by transformation of the plasmid pVP001 linearized with NotI together with pLWA25. Similar to the deletion, two homologous overlap fragments were generated using PCR with primers *v006_GRE3_US_Epimerase* and *v007_GRE3_DS_Epimerase* together with *87R* and *84F* (Additional file 1: Table S3). The resulting triple-fragment double homologous recombination resulted in the generation of a strain with *GRE3* replaced by xylose epimerase (TMBVP1105).

TMBVP1005 and TMBVP1105 were further modified by introducing genes from the xylose degradation pathways consisting of a xylose isomerase (XI pathway) or a xylose reductase and xylitol dehydrogenase (XR/XDH pathway) together with a xylulose kinase (XK).

#### Introduction of xylose pathways

For the introduction of the XR/XDH pathway, three copies of the *XR-XDH-XK* encoding gene construct were introduced into TMBVP1005 and TMBVP1105 by transforming the plasmids pLWA31, pLWA32 and pLWA33 linearized with NotI together with the pCfB3053 gRNA to obtain TMBRP024 and TMBRP025, respectively. For the XR/XDH strain containing xylose epimerase, but with *GRE3* intact (TMBRP033), TMB3755 was transformed as described for TMBVP1105 with triple-fragment double homologous recombination.

Three different XI variants were used in this study: one from *Lachnoclostridium phytofermentans* (ClosXI), another one from *Piromyces* sp*.* strain E2 (PiroXI), and a third one isolated from *Parabacteroides* spp. (ParaXI). For each of these XI variants, two copies of the *XI-XK* gene construct were initially introduced into TMBVP1005 and TMBVP1105 by transforming the plasmids pRP021 and pRP023 for ClosXI, pRP024 and pRP026 for ParaXI and pRP027 and pRP029 for PiroXI. The generated strains were named TMBRP016-021.

A second generation of XI strains were generated to increase the gene copy number by introducing a multicopy plasmid containing the respective XI gene variant and were named TMBRP026-031.

### Aerobic shake flask cultivations

The XI strains TMBRP026-031 were incubated in 50-mL Falcon tubes containing 5 mL YPX medium supplemented with 100 mg L^−1^ nourseothricin to maintain the XI gene-containing plasmids for 42 h. The cells were recovered, washed twice with water and used for inoculation of 250-mL baffled shake flasks containing 25 mL YNB medium supplemented with 50 g L^−1^ xylose and 100 mg L^−1^ nourseothricin. Samples were taken every 24 h for 192 h.

Similarly, the XR/XDH strains were precultured in 50-mL Falcon tubes containing 5 mL YPX medium for 16 h, before being washed twice with water and inoculated in 250-mL baffled shake flasks containing 25 mL YNB medium supplemented with 50 g L^−1^ xylose. Samples were taken every 24 h during a 144-h cultivation, with an additional sample at 78 h.

All samples were analyzed for OD_620nm_ and extracellular metabolite concentrations by HPLC.

### Anaerobic cultivations

The strains were cultivated overnight in 50-ml Falcon tubes containing 5 mL YNB medium supplemented with 40 g L^−1^ glucose. The cells were recovered and washed twice with water before being used for inoculation of 160-ml serum vials containing 50 mL of YNB supplemented with 50 g L^−1^ xylose. The serum vials were flushed with N_2_ for at least half an hour prior to cultivation and maintained sealed with rubber stoppers. The incubation was performed at 30 °C and 180 rpm. Samples were collected every 3 h for the first 15 h and every 24 h after that for OD_620nm_, HPLC and flow cytometry measurements.

### Flow cytometry

Flow cytometry measurements were performed using a MACSQuant VYB (Miltenyi Biotec, Germany) equipped with three excitation lasers at 405 nm, 488 nm and 561 nm and eight emission channels. Samples were diluted with phosphate-buffered saline (PBS) at pH 7.4 to OD_620nm_ < 1.0 when necessary, stained with 10 µg/mL propidium iodine (PI) and incubated for 10 min. A total of 20,000 events were collected per sample at a flow rate of 25 µL/min. To avoid background noise, a threshold of 1.0 in side-scatter area (SSC-A) was applied. Data analysis was performed using FlowJo™ v10.8.1 software (BD Life Sciences).

### Analytical methods

Biomass concentrations was determined by optical density measurements (OD_620nm_) using an Ultrospec 2100 pro UV/Visible spectrophotometer (Amersham Biosciences, Buckinghamshire, United Kingdom). Cell dry weight (CDW) measurements were performed by passing 5 mL of culture through a pre-weighed and dried 0.45 µm paper filter, the filter was then dried in a microwave for 10 min and placed in a desiccation chamber for 2 days prior to being weighed again.

Concentrations of extracellular metabolites such as glucose, xylose, xylitol, glycerol, acetate and ethanol were quantified using a Waters HPLC system (Milford, USA) equipped with a Phenomenex Rezex ROA-Organic Acid column operating at 60 °C. Isocratic 5 mM sulfuric acid was used as mobile phase was isocratic 5 mM sulfuric acid and the flow rate was maintained at 0.6 mL/min.

### Supplementary Information

**Additional file 1: Table S1.** Maximum specific growth rates. Maximum specific growth rates (µ_max_) (h^−1^) obtained for the different strains used in this study under aerobic and anaerobic growth using minimal medium (YNB) supplemented with 50 g/L of xylose. Nd: not determined. **Table S2.** Ethanol yields. Ethanol yield (g/g xylose) obtained for the different strains used in this study under anaerobic growth using minimal medium (YNB) supplemented with 50 g/L of xylose. **Table S3.** List of primers used in this study. Lower case letters indicate the segment annealing to a gene whereas upper case letters correspond to primer tails. **Figure S1.** Final growth measurements for anaerobic cultivations of XI and XR/XDH strains. **A** Optical density at 620 nm and **B** cell dry weight (g/L) after 144 h of anaerobic cultivation in serum vials containing YNB supplemented with 50 g/L xylose. **Figure S2.** Aerobic cultivation of XR/XDH strains on glucose. Optical density at 620nm over time during aerobic cultivation in 250 mL baffled shake flasks containing YNB medium supplemented with 20 g/L glucose. Biological replicates were performed.

## Data Availability

The datasets used and/or analyzed during the current study are available from the corresponding author on reasonable request. The flow cytometry data can also be accessed via FlowRepository.
