# Supplementary material for: Impact of xylose epimerase on sugar assimilation and sensing in recombinant Saccharomyces cerevisiae carrying different xylose-utilization pathways
Source: Biotechnol Biofuels Bioprod. 2023 Nov 6;16:168. doi: 10.1186/s13068-023-02422-z (PMC10629123; doi:10.1186/s13068-023-02422-z)
Supplement: Supplementary file 1 — Additional file 1: Table S1. Maximum specific growth rates. Maximum specific growth rates (µmax) (h−1) obtained for the different strains used in this study under aerobic and anaerobic growth using minimal medium (YNB) supplemented with 50 g/L of xylose. Nd: not determined. Table S2. Ethanol yields. Ethanol yield (g/g xylose) obtained for the different strains used in this study under anaerobic growth using minimal medium (YNB) supplemented with 50 g/L of xylose. Table S3. List of primers used in this study. Lower case letters indicate the segment annealing to a gene whereas upper case letters correspond to primer tails. Figure S1. Final growth measurements for anaerobic cultivations of XI and XR/XDH strains. A Optical density at 620 nm and B cell dry weight (g/L) after 144 h of anaerobic cultivation in serum vials containing YNB supplemented with 50 g/L xylose. Figure S2. Aerobic cultivation of XR/XDH strains on glucose. Optical density at 620nm over time during aerobic cultivation in 250 mL baffled shake flasks containing YNB medium supplemented with 20 g/L glucose. Biological replicates were performed. [file 13068_2023_2422_MOESM1_ESM.docx]

Additional file for:

Impact of xylose epimerase on sugar assimilation and sensing in recombinant *Saccharomyces cerevisiae* carrying different xylose-utilization pathways

Viktor C. Persson^‡1^, Raquel Perruca Foncillas^‡1^, Tegan R. Anderes^1^, Clément Ginestet^1^, Marie Gorwa‑Grauslund^1^

^1^Division of Applied Microbiology, Department of Chemistry, Lund University, Lund, Sweden.

^‡^Authors contributed equally.

Email: [marie-francoise.gorwa@tmb.lth.se](mailto:marie-francoise.gorwa@tmb.lth.se)

**Table S1. Maximum specific growth rates**. Maximum specific growth rates (µ_max_) (h^-1^) obtained for the different strains used in this study under aerobic and anaerobic growth using minimal medium (YNB) supplemented with 50 g/L of xylose. Nd: not determined.

| Strain name | Aerobic YNB+X50 | Anaerobic YNB+X50 |
| --- | --- | --- |
| TMB3755 (XR/XDH) | 0.045 ± 0.002 | nd |
| TMBRP033 (epimerase, XR/XDH) | 0.045 ± 0.001 | nd |
| TMBRP024 (gre3Δ, XR/XDH) | 0.052 ± 0.004 | 0.100 ± 0.003 |
| TMBRP025 (gre3:epimerase, XR/XDH) | 0.047 ± 0.002 | 0.098 ± 0.012 |
| TMBRP026 (gre3Δ, ClosXI) | 0.087 ± 0.000 | 0.049 ± 0.011 |
| TMBRP027 (gre3::epimerase, ClosXI) | 0.000 | 0.047 ± 0.000 |
| TMBRP028 (gre3Δ; ParaXI) | 0.081 ± 0.004 | nd |
| TMBRP029 (gre3:epimerase; ParaXI) | 0.085 ± 0.004 | nd |
| TMBRP030 (gre3Δ; PiroXI) | 0.039 ± 0.062 | nd |
| TMBRP031 (gre3:epimerase; PiroXI) | 0.029 ± 0.046 | nd |

**Table S2.** **Ethanol yields**. Ethanol yield (g/g xylose) obtained for the different strains used in this study under anaerobic growth using minimal medium (YNB) supplemented with 50 g/L of xylose.

| Strain name | Ethanol yield (g/g xylose) |
| --- | --- |
| TMBRP024 (gre3Δ, XR/XDH) | 0.37 ± 0.009 |
| TMBRP025 (gre3:epimerase, XR/XDH) | 0.38 ± 0.002 |
| TMBRP026 (gre3Δ, ClosXI) | 0.18 ± 0.068 |
| TMBRP027 (gre3::epimerase, ClosXI) | 0.38 ± 0.003 |

**Table S3.** **List of primers used in this study**. Lower case letters indicate the segment annealing to a gene whereas upper case letters correspond to primer tails.

| Primer name | Sequence (5´to 3´) |
| --- | --- |
| TEF1p_AscI_f | GCTGGCGCGCCGCTatagcttcaaaatgtttctactcc |
| XI-XK_OE_PCR_r | CCCAGATCGTGATTTTTGTTATCCTGCAGtattcgaactgccc |
| PiroXI_SfaAI_f | ATGCGTGCGATCGCTTAatggctaaggaatattttcc |
| PiroXI_MreI_r | ATGCGTCGCCGGCGTTCttactgatacattgcaacaa |
| TEF1p_BamHI | ATGGCTGGATCCCACatagcttcaaaatgtttcta |
| GPM1t_NotI | TACAAGGCGGCCGCCACtattcgaactgcccattc |
| v009_GRE3_DS_Blank | gatgaagattagcacaattcagaaagcgtacttactgccatactcgaatgGCACTAAA TGCCAACATCAG |
| 87R | GCTTTGCTCTCTTGGTGTCT |
| 84F | TGATGAATTCGTAGACGCAG |
| v008_GRE3_US_Blank | cattcgagtatggcagtaagtacgctttctgaattgtgctaatcttcatcTCTTGAAGGA TTGCACTGAC |
| v006_GRE3_US_Epimerase | ACTCTTTCCTTACTCACGAGTAATTCTTGGttcGAATTCactg gccgtcgTCTTGAAGGATTGCACTGAC |
| v007_GRE3_DS_Epimerase | GTTTTGGGACGCTCGAAGGCTTTAATTTGCtatCTGCAGaggc ctgcatgGCACTAAATGCCAACATCAG |


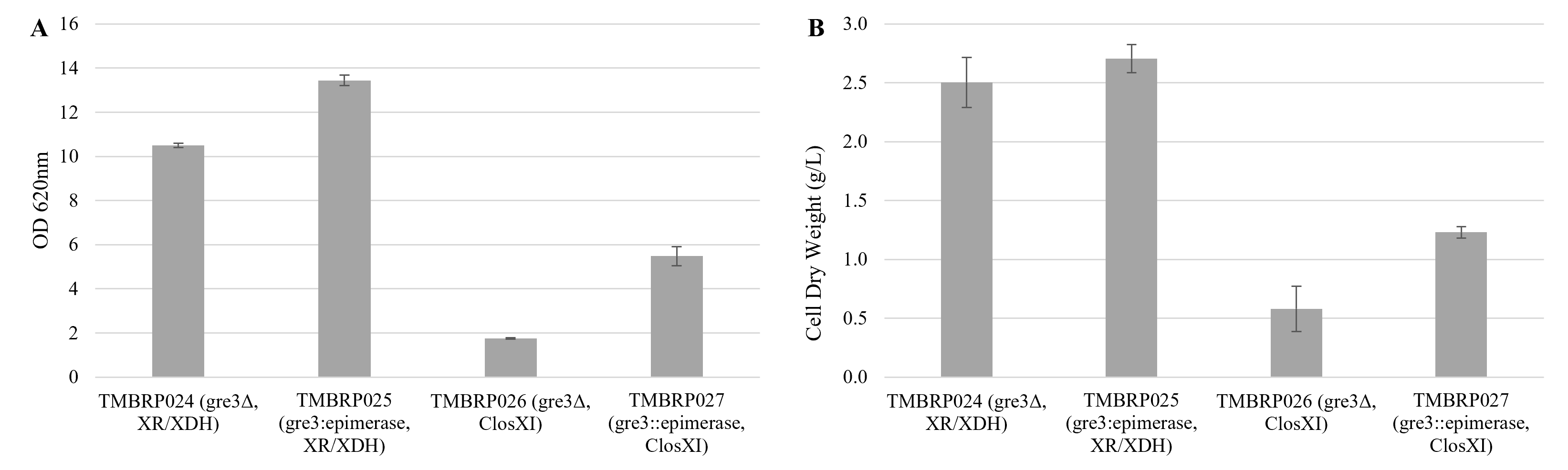


**Fig. S1 Final growth measurements for anaerobic cultivations of XI and XR/XDH strains**. (A) Optical density at 620 nm and (B) cell dry weight (g/L) after 144 hours of anaerobic cultivation in serum vials containing YNB supplemented with 50 g/L xylose.


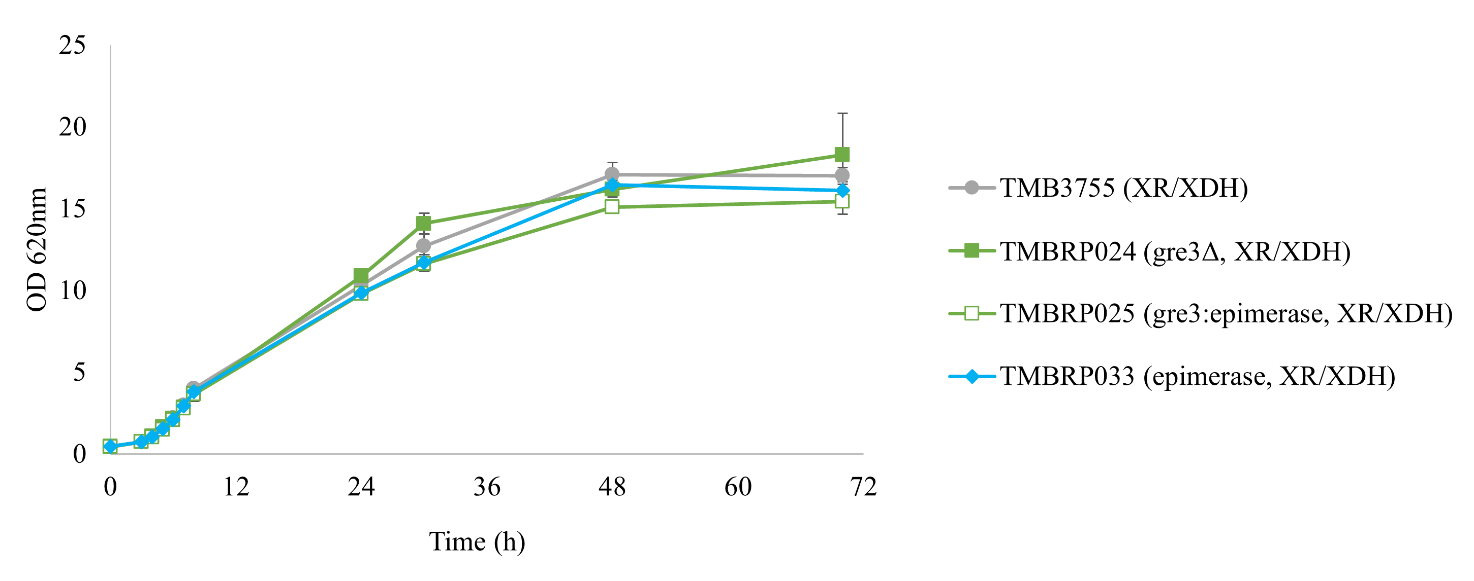


**Fig. S2** **Aerobic cultivation of XR/XDH strains on glucose**. Optical density at 620nm over time during aerobic cultivation in 250mL baffled shake flasks containing YNB medium supplemented with 20 g/L glucose. Biological replicates were performed.
